# Regional estimates of noncommunicable diseases associated risk factors among adults in India: results from National Noncommunicable Disease Monitoring Survey

**DOI:** 10.1186/s12889-022-13466-5

**Published:** 2022-05-30

**Authors:** Thilagavathi Ramamoorthy, Sravya Leburu, Vaitheeswaran Kulothungan, Prashant Mathur

**Affiliations:** Indian Council of Medical Research – National Centre for Disease Informatics and Research, Nirmal Bhawan-ICMR Complex (II Floor), Poojanahalli, Kannamangala Post, Bengaluru, Karnataka 562 110 India

**Keywords:** Epidemiology, India, Noncommunicable diseases, Risk factors, Regions

## Abstract

**Background:**

This study describes regional differences and determinants on key noncommunicable disease (NCD) risk factors in adults from the National NCD Monitoring Survey (NNMS) across six geographic regions of India.

**Methods:**

The NNMS was a cross-sectional multistage cluster survey conducted in 2017–18, on a representative sample of 300 urban and 300 rural primary sampling units (PSU) covering 20 households per PSU. One adult aged 18–69 years per household was selected using the KISH grid. Globally standard survey tools were adapted for data collection. To arrive at regional estimates, the country was divided into six regions (south, north, central, west, east and northeast) based on the distribution of a national sample. The results are presented as proportion with 95% confidence intervals (CI). Univariable and multivariable logistic regression analyses were performed to identify NCD risk factor determinants significant in the regions. A *p*-value < 0.05 was considered for statistical significance.

**Results:**

The overall survey response rate was 96.3%. The prevalence of current tobacco (45.7%) and alcohol use (22.3%) was significantly high in the northeast region. The highest proportion of adults from northern India showed low levels of physical activity (49.6%). The prevalence of metabolic risk factors — obesity (12.5%), raised fasting blood glucose (21.2%) and raised blood pressure (35.6%) was highest in south India. The prevalence of raised blood pressure was high in north India (35.2%) similar in proportion to south India. Clustering of ≥3 risk factors (50.1%) and ten-year CVD risk of ≥30% or with existing CVD (18.1%) was highest in south India when compared to other regions. Older age, urban residents, alcohol consumption and overweight/obesity were significantly associated with higher odds of raised blood pressure and raised fasting blood glucose.

**Conclusion:**

The NNMS presents variations in NCD risk factors within the regions of India. It contributes to robust evidence for strengthening interventions and monitoring the progress in reducing NCDs and their associated risk factors.

**Supplementary Information:**

The online version contains supplementary material available at 10.1186/s12889-022-13466-5.

## Background

The disease epidemiology in India has transitioned within the past 2 decades from infectious diseases, undernutrition, maternal and childhood diseases to the increasing burden of noncommunicable diseases (NCDs) [1]. NCDs contributed to 65% of all deaths in the country in 2019 [2]. They are primarily driven by the high prevalence of major preventable risk factors — tobacco use, consumption of alcohol, unhealthy dietary practices, lack of sufficient physical activity, overweight/obesity, hypertension, diabetes and hyperlipidemia. Their clustering in individuals contributes to the major NCDs like cardiovascular diseases (CVDs), cancer, diabetes and stroke [3]. While these epidemiological transitions are seen across the globe, the heterogeneity within India makes it unique and challenging.

In 2013, the Government of India adopted the Global NCD Monitoring Targets and formulated the national specific NCD targets (10) and indicators (21) to be achieved by 2025 [4]. Though there have been other large national and sub-national surveys being periodically conducted in India, like the National Family Health Survey (NFHS), Global Adult Tobacco Survey (GATS), Magnitude of Substance Use study, India Diabetes study, Longitudinal Ageing Study in India, and a few state-specific NCD risk factor surveys [1, 5–13] they lack in providing a complete national NCD risk factor profile aligning with the NCD monitoring framework [4]. In addition, these surveys differ in their study objectives, methodology, age categories and indicator definitions. Fulfilling these set NCD targets, the National NCD Monitoring Survey (NNMS) provided robust evidence on specific NCD risk factors [3, 14–16].

India is the world’s second-most populous country with 28 states and 8 union territories (UTs). All Indian states and UTs are categorized into six administrative zones/regions namely north, west, central, south, east and northeast [17, 18]. Every region in India is large and different in their cultural and lifestyle practices, economic and social development; socio-demographic profile; and disease epidemiology. Furthermore, NCDs and their associated risk factors are emerging as a major concern across all the regions irrespective of their economic and health systems profile [1, 5, 19, 20]. There is a need to study the regional-level NCD risk factor profile in such a large and diverse country. Such assessments are essential for planning and optimizing NCD prevention and control interventional strategies. This paper aimed at generating region-wise prevalence and determinants of key NCD risk factors among adults aged 18–69 years, utilizing the primary data from the National Noncommunicable Disease Monitoring Survey (2017–18).

## Methods

The NNMS was the first comprehensive and NCD specific national community-based, cross-sectional survey conducted from October 2017 to April 2018 to estimate key NCD indicators among adults (18–69 years), adolescents (15–17 years) and national health system preparedness to NCDs in India as identified in the National NCD Monitoring Framework and Action Plan [3, 14–16]. This survey was coordinated by the Indian Council of Medical Research (ICMR) - National Centre for Disease Informatics and Research (NCDIR) through a collaborating network of ten implementing research institutes of repute across the country [3, 14–16].

### Sampling design and size

**Fig. 1:**
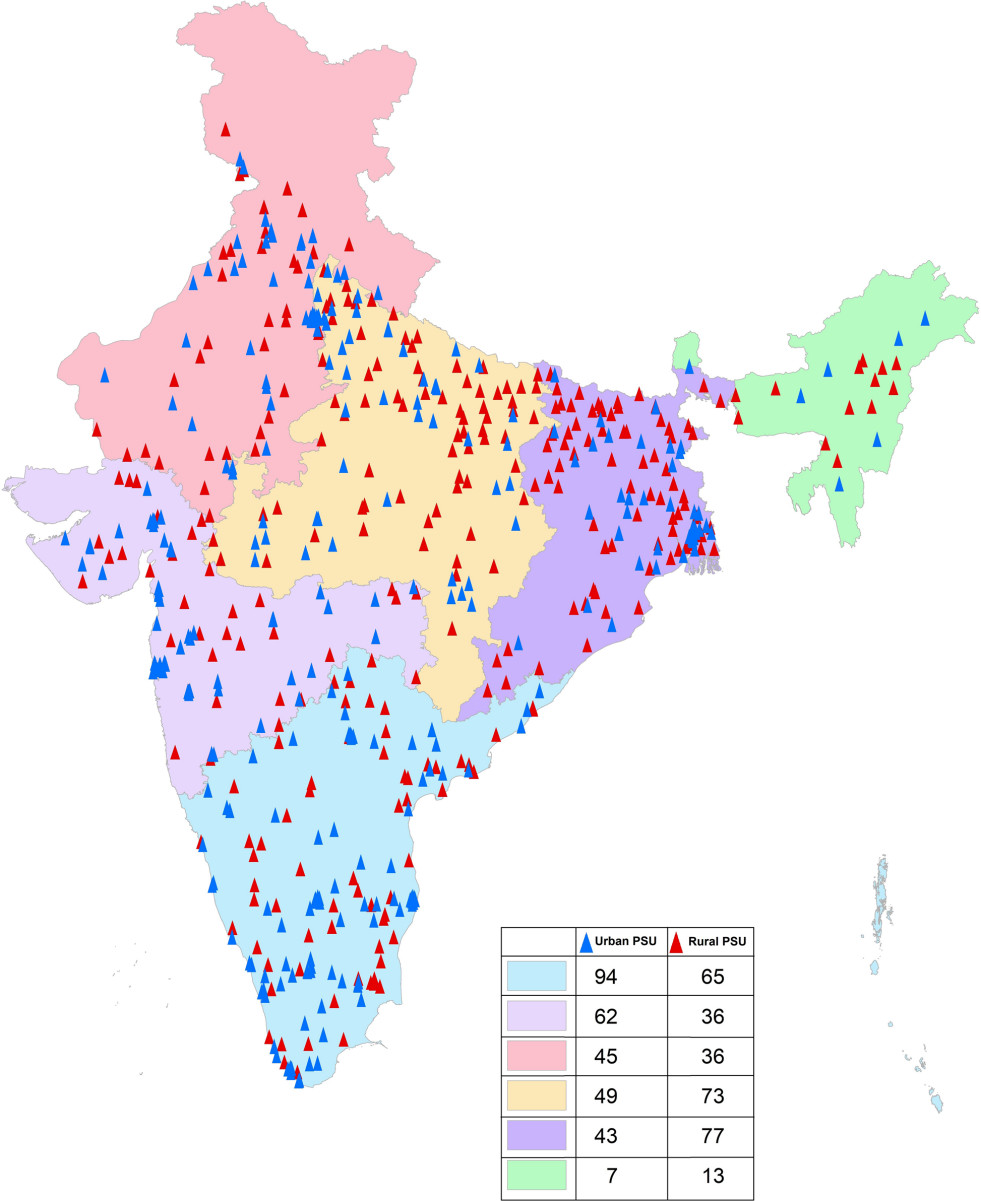
Geographical distribution of sampled clusters under NNMS 2017–18

The NNMS survey adopted a multistage cluster sampling design in both urban and rural areas. A nationally representative sample of 12,000 adults from 600 primary sampling units (PSUs) equally distributed as 300 wards (an administrative unit of the city) in urban areas and 300 villages in rural areas were selected through probability proportional to size sampling method [3, 14–17]. (Fig. 1). A series of publications from NNMS provides detailed survey methodology of the survey [3, 14–16].

### Study tools

The survey adopted standard study tools for adults [3, 14–16] — WHO STEPwise approach to noncommunicable disease risk factor surveillance (WHO-STEPS) [21], Integrated disease surveillance project (IDSP)-NCD risk factor survey [12], and GATS, India [22]. The study tools were developed in English language and translated into eleven Indian local languages. The translated questionnaires were validated using the forward-backward translation method. The study interviews were conducted using the hand-held tablets through an Open Data Kit android offline application [3, 23, 24].

### Ethical approval and consent

The study had obtained ethical clearance from the institutional ethics committee at ICMR-NCDIR and, all ten implementing agencies obtained ethics approval separately from their respective institutional ethics committees. The eligible adult participants were briefed about the objective and purpose of the survey. All adults were enrolled on the study after obtaining informed written consent. Pamphlets in local languages with health promotion and NCD prevention messages were provided to all the participants. Those exposed to both behavioural and metabolic risk factors of NCDs were referred to the nearest public health facility for further management.

### Indicators and definitions

The present analysis used the survey information collected on behavioural risk factors namely tobacco use, alcohol consumption and physical activity and metabolic risk factors — overweight (including obesity), central obesity, raised fasting blood glucose and raised blood pressure. Standard definitions were used to derive these indicators (Supplementary Table 1). The clustering of risk factors was defined as the presence of three or more risk factors — tobacco use, inadequate fruits and/or vegetable intake, insufficient physical activity, overweight (including obesity) (BMI ≥25.0Kg/m^2^), raised blood pressure and raised fasting blood glucose including those on medication [25]. The ten-year CVD risk assessment was calculated as per WHO–International Society of Hypertension Cardiovascular Disease Risk Prediction Charts (2007) for South East Asia region based on sex, age (40–69 years), systolic blood pressure, current smoked tobacco use and diabetes (previously diagnosed/fasting blood glucose concentration ≥ 126 mg/dl) [26]. Regions were categorized based on the PSUs covered in the state/UT for the survey as — South (Andhra Pradesh, Telangana, Karnataka, Kerala and Tamil Nadu), West (Gujarat and Maharashtra), North (Jammu and Kashmir, Himachal Pradesh, Punjab, Chandigarh, Uttarakhand, Haryana, Delhi and Rajasthan), Central (Uttar Pradesh, Chhattisgarh and Madhya Pradesh), East (Bihar, West Bengal, Jharkhand and Orissa) and Northeast (Sikkim, Nagaland, Manipur, Mizoram and Assam) based on the population proportion covered in the survey [6, 17, 18].

### Data management and statistical analysis

Cleaned unweighted data were analysed using IBM SPSS software *(Version 27.0*; *IBM Corp, Armonk, NY, USA)*. The results by region are presented as numbers and proportions by place of residence, sex and age groups. The Pearson’s Chi-square analysis was performed to find the significant difference between the regions. Multivariable logistic regression analysis was done to identify the NCD risk factors significant in the regions and adjusted odds ratio (aOR) was calculated with a 95% confidence interval (CI). A *p*-value < 0.05 was considered for statistical significance. In addition, this study also compared the regional prevalence of risk factors with their Human Development Index (HDI) and its components (health index, education index and income index) [27, 28] to study the socio-economic impact. The state-specific HDI and its component indices were obtained for the year 2017–18 from the United Nations Development Programme [27, 28] and these numbers were pooled to arrive at the respective regional average index measures. All results are provided as tables, figures and supplementary tables.

## Results

Out of 12,000 selected households, 11,139 households completed the household questionnaires and among these, a total of 10,659 adults (5048 adults (47.4%) from urban areas and 5611 adults (52.6%) from rural areas) completed the adult interviews. The overall response rate of the survey was 96.3%. Table 1 shows the region-wise distribution of the adult population as per the 2011, Census of India, NNMS sample distribution and the response rates at the level of household and adults.

**Table 1:** Distribution of survey sample and response rates (Percentage)

Region	Census 2011 adult population	Survey Sample distribution (%)	Response rate (%)	
	(18–69 years) (%)		Household	Adult
South	22.9	26.5	95.9	97.1
West	15.3	16.3	90.4	92.1
North	13.9	13.5	92.3	93.7
Central	22.6	20.3	98.0	96.2
East	21.6	20.0	98.4	99.6
Northeast	3.7	3.3	98.5	100.0

### Prevalence of behavioural risk factors

#### Tobacco use

**Fig. 2:**
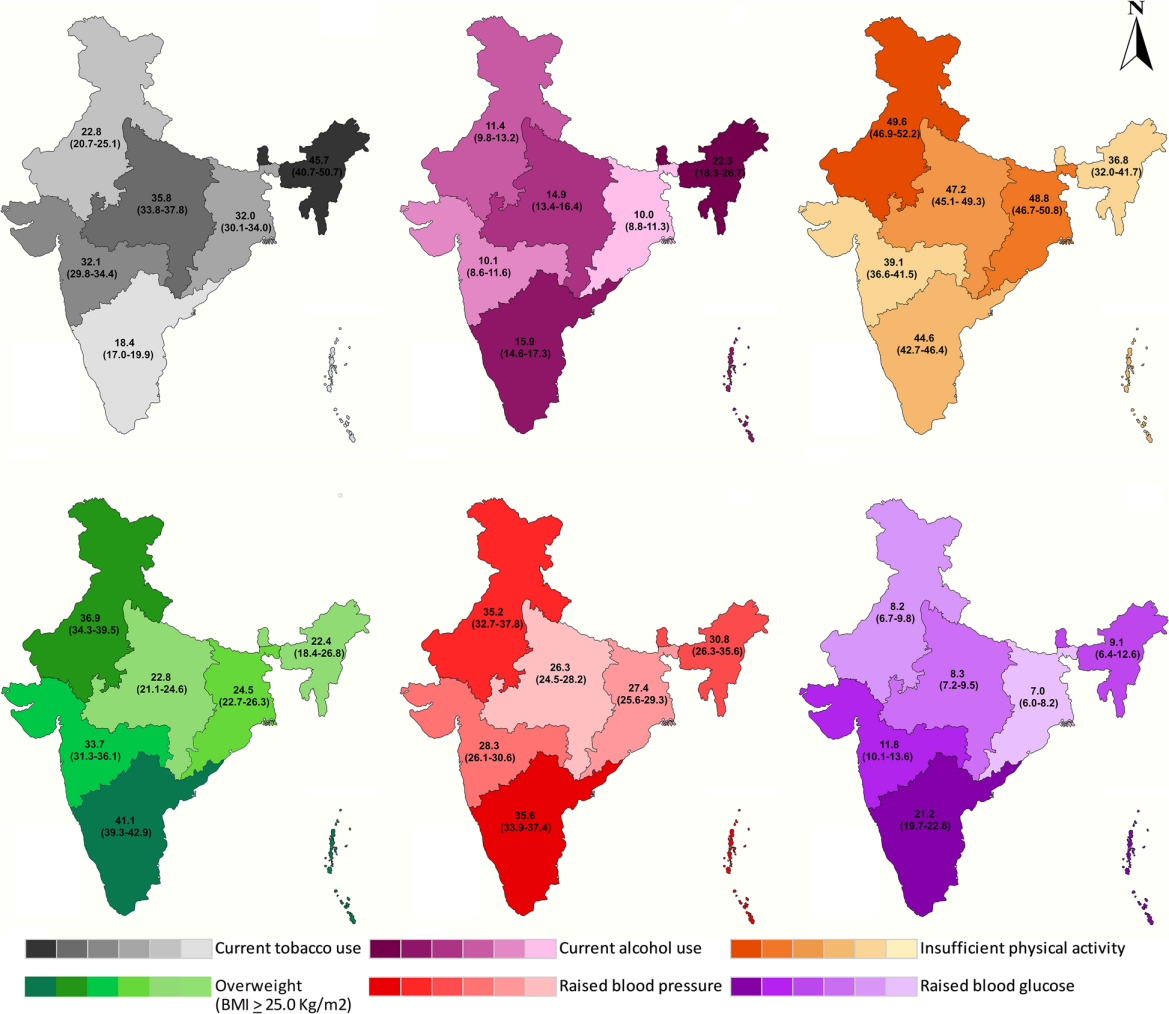
Prevalence of NCD risk factors among adults aged 18–69 years in India by region

Northeast region showed significantly highest prevalence of current tobacco use in any form [45.7% (95% CI: 40.7–50.7)], daily use of smoked tobacco [15.5% (95% CI: 12.1–19.4)] as well as smokeless forms [33.6% (95% CI: 29.0–38.4)] than other regions of the country. The lowest prevalence of current tobacco use was in southern region [18.4% (95% CI: 17.0–19.9)] (Fig. 2 and supplementary Table 2).

The current use of smoked and/or smokeless tobacco was lowest [6.4% (95% CI: 4.8–8.3)] among women in the northern region and highest [61.8% (95% CI: 54.9–68.3)] among men in the northeast. Adults aged 30–69 years showed the highest consumption of tobacco, with predominance in the adults between 30 and 49 years from the northeast [50.5% (95% CI: 43.4–57.7)]. Young adults aged 18–29 years from southern India [12.6% (95% CI: 10.2–15.4)] showed the lowest consumption of tobacco. The western region presented a low prevalence of daily use of smoked tobacco [4.1% (95% CI: 3.2–5.1)], while daily smokeless tobacco use was lower in the south [9.1% (95% CI: 8.0–10.2)] (Table 2 and supplementary Table 2).

**Table 2:** Prevalence of behavioural risk factors associated with NCDs among adults aged 18–69 years in India by place of residence, sex, age group and region

Behavioural risk factors	Place of residence		Sex		Age group		
	Urban	Rural	Men	Women	18–29 Years	30–49 Years	50–69 Years
	n (%)	n (%)	n (%)	n (%)	n (%)	n (%)	n (%)
**Current tobacco use (smoke and/or smokeless variety)** (in last 12 months)							
South	241 (14.7)	284 (23.6)	392 (32.5)	133 (8.1)	78 (12.6)	246 (18.8)	201 (21.8)
West	251 (27.3)	247 (39.1)	385 (49.2)	113 (14.7)	87 (21.7)	249 (34.3)	162 (38.0)
North	149 (21.3)	160 (24.5)	260 (44.1)	49 (6.4)	65 (17.1)	164 (26.6)	80 (22.5)
Central	243 (28.6)	559 (40.2)	636 (60.1)	166 (14.0)	179 (26.6)	399 (38.5)	224 (42.0)
East	225 (28.1)	506 (34.2)	558 (55.9)	173 (13.5)	139 (21.7)	365 (34.4)	227 (39.1)
Northeast	63 (48.8)	111 (44.0)	123 (61.8)	51 (28.0)	27 (32.5)	93 (50.5)	54 (47.4)
***p value***	***< 0.001***	***< 0.001***	***< 0.001***	***< 0.001***	***< 0.001***	***< 0.001***	***< 0.001***
**Current daily smoked tobacco use** (in last 12 months)							
South	110 (6.7)	100 (8.3)	197 (16.3)	13 (0.8)	25 (4.1)	93 (7.1)	92 (10.0)
West	42 (4.6)	21 (3.3)	63 (8.0)	0 (0.0)	8 (2.0)	28 (3.9)	27 (6.3)
North	58 (8.3)	77 (11.8)	124 (21.1)	11 (1.4)	14 (3.7)	68 (11.0)	53 (14.9)
Central	70 (8.2)	175 (12.6)	229 (21.6)	16 (1.4)	33 (4.9)	121 (11.7)	91 (17.1)
East	66 (8.2)	114 (7.7)	173 (17.3)	7 (0.5)	32 (5.0)	78 (7.4)	70 (12.0)
Northeast	28 (21.7)	31 (12.3)	49 (24.6)	10 (5.5)	6 (7.2)	35 (19.0)	18 (15.8)
***p value***	***< 0.001***	***< 0.001***	***< 0.001***	***< 0.001***	*0.115*	***< 0.001***	***< 0.001***
**Current daily smokeless tobacco use** (in last 12 months)							
South	99 (6.0)	159 (13.2)	150 (12.4)	108 (6.6)	38 (6.2)	116 (8.8)	104 (11.3)
West	164 (17.8)	186 (29.5)	256 (32.7)	94 (12.2)	58 (14.5)	184 (25.4)	108 (25.4)
North	77 (11.0)	72 (11.0)	119 (20.2)	30 (3.9)	38 (10.0)	84 (13.6)	27 (7.6)
Central	153 (18.0)	384 (27.6)	405 (38.2)	132 (11.2)	129 (19.2)	268 (25.8)	140 (26.3)
East	158 (19.7)	340 (23.0)	361 (36.2)	137 (10.7)	83 (13.0)	258 (24.3)	157 (27.0)
Northeast	41 (31.8)	87 (34.5)	85 (42.7)	43 (23.6)	17 (20.5)	73 (39.7)	38 (33.3)
***p value***	***< 0.001***	***< 0.001***	***< 0.001***	***< 0.001***	***< 0.001***	***< 0.001***	***< 0.001***
**Second-hand tobacco smoke exposure at home** (in past 30 days)							
South	307 (18.7)	262 (21.8)	277 (23.0)	292 (17.8)	140 (22.7)	269 (20.5)	160 (17.4)
West	238 (25.8)	239 (37.9)	267 (34.1)	210 (27.3)	118 (29.4)	240 (33.1)	119 (27.9)
North	215 (30.7)	289 (44.3)	243 (41.3)	261 (34.2)	151 (39.6)	231 (37.5)	122 (34.3)
Central	227 (26.7)	488 (35.1)	437 (41.3)	278 (23.5)	228 (33.9)	328 (31.6)	159 (29.8)
East	298 (37.2)	460 (31.1)	360 (36.1)	398 (31.0)	202 (31.6)	359 (33.8)	197 (33.9)
Northeast	55 (42.6)	98 (38.9)	75 (37.7)	78 (42.9)	41 (49.4)	69 (37.5)	43 (37.7)
***p value***	***< 0.001***	***< 0.001***	***< 0.001***	***< 0.001***	***< 0.001***	***< 0.001***	***< 0.001***
**Current alcohol use** (in last 12 months)							
South	251 (15.3)	203 (16.9)	418 (34.7)	36 (2.2)	86 (13.9)	232 (17.7)	136 (14.8)
West	88 (9.6)	68 (10.8)	150 (19.2)	6 (0.8)	23 (5.7)	87 (12.0)	46 (10.8)
North	86 (12.3)	68 (10.4)	150 (25.5)	4 (0.5)	30 (7.9)	86 (14.0)	38 (10.7)
Central	133 (15.6)	200 (14.4)	326 (30.8)	7 (0.6)	95 (14.1)	176 (17.0)	62 (11.6)
East	60 (7.5)	168 (11.3)	201 (20.1)	27 (2.1)	62 (9.7)	118 (11.1)	48 (8.3)
Northeast	37 (28.7)	48 (19.0)	70 (35.2)	15 (8.2)	16 (19.3)	52 (28.3)	17 (14.9)
***p value***	***< 0.001***	***< 0.001***	***< 0.001***	***< 0.001***	***< 0.001***	***< 0.001***	***0.005***
**Heavy episodic drinking**^a^							
South	98 (6.0)	70 (5.8)	167 (13.8)	1 (0.1)	24 (3.9)	100 (7.6)	44 (4.8)
West	30 (3.3)	27 (4.3)	56 (7.2)	1 (0.1)	3 (0.7)	38 (5.2)	16 (3.8)
North	39 (5.6)	28 (4.3)	67 (11.4)	0 (0.0)	12 (3.1)	39 (6.3)	16 (4.5)
Central	49 (5.8)	82 (5.9)	129 (12.2)	2 (0.2)	32 (4.8)	76 (7.3)	23 (4.3)
East	23 (2.9)	49 (3.3)	61 (6.1)	11 (0.9)	20 (3.1)	36 (3.4)	16 (2.8)
Northeast	12 (9.3)	16 (6.3)	27 (13.6)	1 (0.5)	2 (2.4)	20 (10.9)	6 (5.3)
***p value***	***< 0.001***	**0.009**	***< 0.001***	***< 0.001***	***0.019***	***< 0.001***	***0.488***
**Insufficient physical activity**^b^							
South	808 (49.8)	449 (37.5)	422 (35.7)	835 (50.9)	262 (43.6)	546 (42.0)	449 (48.9)
West	402 (44.6)	190 (30.9)	264 (34.8)	328 (43.3)	133 (34.3)	272 (38.2)	187 (45.0)
North	408 (58.2)	262 (40.2)	248 (42.2)	422 (55.2)	175 (46.1)	281 (45.6)	214 (60.1)
Central	507 (59.9)	543 (39.4)	403 (38.6)	647 (54.7)	307 (46.3)	451 (43.8)	292 (54.8)
East	448 (56.5)	656 (44.6)	261 (26.7)	843 (65.7)	311 (49.2)	485 (46.1)	308 (53.1)
Northeast	55 (43.7)	84 (33.3)	44 (22.3)	95 (52.5)	31 (37.8)	59 (32.4)	49 (43.0)
***p value***	***< 0.001***	***< 0.001***	***< 0.001***	***< 0.001***	***< 0.001***	***0.001***	***< 0.001***

^a^Heavy episodic drinking constitutes those who reported drinking ≥6 standard drinks (equivalent to 60 g of pure alcohol or ethanol) in a single drinking occasion in the last 30 days of the interview

^b^Insufficient physical activity constitutes those who engaged in < 150 minutes of moderate-intensity physical activity per week OR < 75 minutes of vigorous-intensity physical activity per week OR an equivalent combination of moderate- and vigorous-intensity physical activity accumulating < 600 MET – minutes per week

More than one-fifth adults across all regions were exposed to second-hand tobacco smoke at home, with the highest in the northeast region [40.2% (95% CI: 35.3–45.1)] (Supplementary Table 2).

#### Alcohol use

The prevalence of current alcohol use was highest in the northeast (22.3%) and lowest in the western (10.1%) and eastern (10.0%) parts of India (Fig. 2).

The highest proportion of men aged between 18 and 69 years from the northeast region reported alcohol consumption [35.2% (95% CI: 28.8–42.0)] in the last 12 months before the survey. The prevalence of heavy episodic drinking was higher among men in the south [13.8% (95% CI:12.0–15.9)] followed by northeast [13.6% (95% CI: 9.3 - 18.8)]. Across the age groups, the highest prevalence (28.3%) of current alcohol use was among the middle-aged adults (30–49 years) from northeast India, while the lowest was among the younger adults (18–29 years) from western India (5.7%) (Table 2 and supplementary Table 2). These findings were statistically significant *(p < 0.001)* at a 5% level of significance.

#### Insufficient physical activity

Almost half adults in the north [49.6% (95% CI: 46.9–52.2)], east [48.8% (95% CI: 46.7–50.8)] and central [47.2% (95% CI: 45.1–49.3)] regions were doing insufficient levels of physical activity in a week. These findings were statistically significant *(p < 0.001).* Across all the six regions, women and urban adults were insufficiently physically active compared to men and rural adults. The highest prevalence of insufficient physical activity was among the older adults (50–69 years) from north India (60.1%). The younger (49.2%) and middle (46.1%) aged adults from eastern India also showed significant insufficient levels of physical activity (Fig. 2, Table 2 and supplementary Table 2).

### Prevalence of metabolic risk factors

Southern India showed significantly highest prevalence of overweight [41.1% (95% CI: 39.3–42.9)], obesity [12.5% (95% CI: 11.3–13.7)], central obesity [49.5% (95% CI: 47.7–51.4)], raised blood pressure [35.6% (95% CI: 33.9–37.4)] and raised fasting blood glucose [21.2% (95% CI: 19.7–22.8)]. The prevalence of raised blood pressure in the north [35.2 (95% CI: 32.7–37.8)] was similar to the prevalence in the south. Northeast India showed the lowest prevalence of overweight (22.4%), obesity (3.7%) and central obesity (29.0%), while raised blood pressure and raised fasting blood glucose was lowest in the central (26.3%) and eastern India (7.0%), respectively (Fig. 2, Table 3 and supplementary Table 3).

**Table 3:** Prevalence of metabolic risk factors associated with NCDs among adults in India aged 18–69 years by place of residence, sex, age group and region

Metabolic risk factors	Place of residence		Sex		Age group		
	Urban	Rural	Men	Women	18–29 Years	30–49 Years	50–69 Years
	n (%)	n (%)	n (%)	n (%)	n (%)	n (%)	n (%)
**Overweight (BMI ≥ 25.0 Kg/m**^**2**^**)**							
South	802 (50.3)	343 (28.8)	427 (36.2)	718 (44.7)	173 (28.8)	582 (45.5)	390 (43.0)
West	376 (43.0)	125 (20.4)	218 (28.6)	283 (39.1)	71 (18.9)	273 (38.9)	157 (38.3)
North	310 (46.5)	168 (26.6)	169 (29.8)	309 (42.4)	77 (21.2)	236 (40.2)	165 (47.7)
Central	312 (37.5)	187 (13.8)	208 (20.0)	291 (25.4)	81 (12.7)	277 (27.2)	141 (26.7)
East	273 (35.4)	270 (18.6)	196 (20.0)	347 (28.1)	107 (17.6)	301 (28.9)	135 (23.6)
Northeast	46 (36.2)	38 (15.3)	41 (20.6)	43 (24.4)	10 (12.5)	50 (27.6)	24 (21.1)
***p value***	***< 0.001***	***< 0.001***	***< 0.001***	***< 0.001***	***< 0.001***	***< 0.001***	***< 0.001***
**Obesity (BMI ≥ 30.0 Kg/m**^**2**^**)**							
South	262 (16.4)	85 (7.1)	106 (9.0)	241 (15.0)	47 (7.8)	177 (13.8)	123 (13.6)
West	110 (12.6)	32 (5.2)	45 (5.9)	97 (13.4)	13 (3.5)	77 (11.0)	52 (12.7)
North	92 (13.8)	51 (8.1)	37 (6.5)	106 (14.5)	20 (5.5)	66 (11.2)	57 (16.5)
Central	88 (10.6)	35 (2.6)	39 (3.8)	84 (7.3)	11 (1.7)	83 (8.1)	29 (5.5)
East	70 (9.1)	38 (2.6)	32 (3.3)	76 (6.1)	19 (3.1)	63 (6.1)	26 (4.5)
Northeast	7 (5.5)	7 (2.8)	5 (2.5)	9 (5.1)	1 (1.3)	8 (4.4)	5 (4.4)
***p value***	***< 0.001***	***< 0.001***	***< 0.001***	***< 0.001***	***< 0.001***	***< 0.001***	***< 0.001***
**Central obesity**^a^							
South	925 (57.8)	457 (38.4)	454 (38.3)	928 (57.7)	180 (30.0)	683 (53.3)	519 (57.2)
West	387 (44.3)	130 (21.2)	232 (30.4)	285 (39.4)	54 (14.4)	277 (39.4)	186 (45.4)
North	400 (59.9)	225 (35.5)	203 (35.6)	422 (57.7)	95 (26.0)	301 (51.0)	229 (66.0)
Central	364 (43.8)	302 (22.3)	247 (23.7)	419 (36.6)	86 (13.5)	367 (35.9)	213 (40.2)
East	341 (44.1)	360 (24.8)	166 (16.9)	535 (43.1)	118 (19.4)	371 (35.6)	212 (36.8)
Northeast	53 (41.7)	56 (22.5)	33 (16.6)	76 (42.9)	14 (17.5)	59 (32.4)	36 (31.6)
***p value***	***< 0.001***	***< 0.001***	***< 0.001***	***< 0.001***	***< 0.001***	***< 0.001***	***< 0.001***
**Raised blood pressure**^b^							
South	616 (37.6)	396 (32.9)	438 (36.5)	574 (35.0)	74 (12.0)	401 (30.8)	537 (58.3)
West	271 (29.8)	163 (26.0)	223 (28.8)	211 (27.8)	43 (10.8)	194 (27.1)	197 (46.7)
North	259 (37.4)	212 (32.9)	229 (39.5)	242 (31.9)	67 (17.8)	197 (32.6)	207 (58.1)
Central	286 (33.8)	300 (21.8)	282 (27.0)	304 (25.8)	77 (11.6)	276 (26.8)	233 (43.9)
East	249 (31.6)	373 (25.2)	262 (26.5)	360 (28.2)	87 (13.7)	269 (25.6)	266 (45.9)
Northeast	45 (34.9)	72 (28.7)	70 (35.2)	47 (26.0)	8 (9.6)	51 (27.9)	58 (50.9)
***p value***	***0.001***	***< 0.001***	***< 0.001***	***< 0.001***	***0.034***	***0.012***	***< 0.001***
**Raised fasting blood glucose**^c^							
South	360 (24.8)	186 (16.6)	223 (20.5)	323 (21.8)	20 (3.7)	204 (17.3)	322 (37.8)
West	117 (14.8)	41 (7.5)	78 (11.5)	80 (12.1)	10 (2.9)	63 (10.1)	85 (22.9)
North	66 (10.9)	32 (5.4)	43 (8.1)	55 (8.2)	4 (1.2)	27 (5.0)	67 (21.0)
Central	89 (11.6)	83 (6.3)	73 (7.5)	99 (9.0)	16 (2.6)	71 (7.4)	85 (16.6)
East	73 (10.5)	72 (5.2)	54 (6.0)	91 (7.8)	10 (1.8)	60 (6.3)	75 (13.8)
Northeast	15 (14.9)	15 (6.6)	18 (10.8)	12 (7.5)	1 (1.5)	14 (8.8)	15 (14.6)
***p value***	***< 0.001***	***< 0.001***	***< 0.001***	***< 0.001***	***0.191***	***< 0.001***	***< 0.001***

^a^Central obesity was defined as having a waist circumference of ≥90 cm in males and ≥ 80 cm in females

^b^Raised blood pressure was when the systolic blood pressure was≥140 mm of Hg and/or diastolic blood pressure ≥ 90 mm of Hg including those on medication for raised BP among adults aged 18–69 years

^c^Raised fasting blood glucose was when the values of fasting blood glucose were ≥ 126 mg/dl including those on medication for raised blood glucose among adults aged 18–69 years

The prevalence of metabolic risk factors other than central obesity and raised fasting blood glucose was lowest in the rural areas of central India. A higher proportion of women were found to be obese than men across all the regions. Across the age groups, older adults had higher metabolic risk factors, with a high prevalence of overweight (47.7%), obesity (16.5%) and central obesity (66.0%) in the north; and raised blood pressure (58.3%) and raised fasting blood glucose (37.8%) being highest in the south (Fig. 2, Table 3 and supplementary Table 3).

### Composite risk assessment

**Fig. 3:**
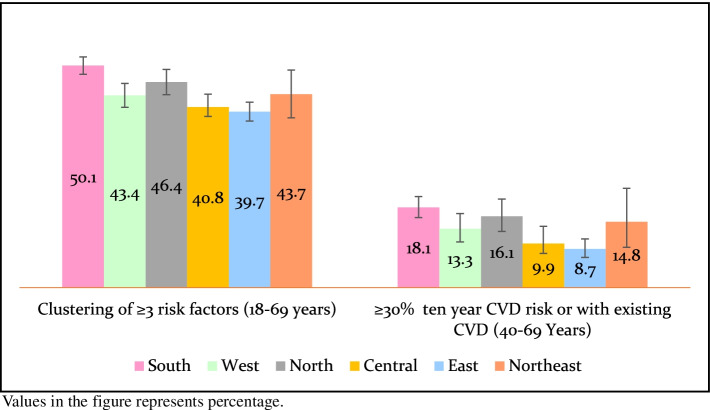
Composite risk assessment among adults aged 18–69 years in India by region

Almost half adults had clustering of three or more risk factors for NCDs in the south [50.1% (95% CI: 48.2–52.0)] and north [46.4% (95% CI: 43.5–49.2)]. A lower proportion of risk factor clustering was seen amongst the rural adults from central India [32.9% (95% CI: 30.4–35.5)]. Similarly, ≥30% (or with existing CVD) of ten-year CVD risk among adults aged 40–69 years was highest in south [18.1% (95% CI: 15.8–20.5)] and north [16.1% (95% CI: 12.7–20.0)]. While 8.7% (95% CI: 6.8–11.0) adults from east and 9.9% (95% CI: 7.7–12.5) from the central region were at lowest risk for the development of CVDs (Fig. 3, supplementary Tables 4 and 5).

### Region-wise determinants of raised blood pressure and raised fasting blood glucose

Older adults (50–69 years) from the northeast (aOR: 9.99) and southern India (aOR: 9.90); adults with overweight including obesity from central India (aOR: 2.74), current alcohol users from the northeast (aOR: 2.31), and men from northern India (aOR: 1.40) had higher odds of risk for raised blood pressure (Table 4)*.*

**Table 4:** Determinants of raised blood pressure and raised fasting blood glucose by region among adults aged 18–69 years in India

	Raised blood pressure						Raised fasting blood glucose					
	South	West	North	Central	East	Northeast	South	West	North	Central	East	Northeast
	aOR (95% CI)	aOR (95% CI)	aOR (95% CI)	aOR (95% CI)	aOR (95% CI)	aOR (95% CI)	aOR (95% CI)	aOR (95% CI)	aOR (95% CI)	aOR (95% CI)	aOR (95% CI)	aOR (95% CI)
**Sex**												
Men	1.09 (0.90–1.33)	0.99 (0.76–1.29)	**1.40 (1.05–1.87)**	1.00 (0.78–1.28)	0.83 (0.66–1.05)	1.21 (0.72–2.06)	1.08 (0.85–1.36)	1.02 (0.69–1.50)	1.07 (0.64–1.81)	0.71 (0.48–1.07)	0.98 (0.64–1.50)	1.29 (0.53–3.13)
Women	Ref						Ref					
**Age group**												
18–29 Years	Ref						Ref					#
30–49 Years	**2.94 (2.23–3.88)**	**2.40 (1.66–3.47)**	**1.87 (1.35–2.60)**	**2.25 (1.69–3.00)**	**2.00 (1.52–2.62)**	**2.96 (1.30–6.72)**	**4.88 (3.03–7.86)**	**2.82 (1.40–5.65)**	**3.33 (1.14–9.74)**	**2.53 (1.42–4.50)**	**3.79 (1.85–7.74)**	
50–69 Years	**9.90 (7.46–13.16)**	**6.07 (4.14–8.89)**	**5.29 (3.72–7.52)**	**5.11 (3.77–6.93)**	**5.29 (3.95–7.06)**	**9.99 (4.29–23.25)**	**15.06 (9.39–24.16)**	**8.01** **(4.02–15.97)**	**16.74 (5.93–47.25)**	**6.98 (3.93–12.39)**	**9.25 (4.54–18.83)**	**2.97 (1.31–6.74)**
**Place of Residence**												
Urban	1.16 (0.97–1.39)	1.08 (0.84–1.40)	1.03 (0.79–1.32)	**1.47 (1.18–1.83)**	1.21 (0.98–1.49)	1.07 (0.64–1.80)	**1.49 (1.20–1.86)**	**1.70 (1.13–2.54)**	**1.75 (1.08–2.85)**	**1.71 (1.21–2.43)**	**1.70 (1.18–2.44)**	**2.26 (1.01–5.20)**
Rural	Ref						Ref					
**Current Tobacco use**												
Yes	1.04 (0.82–1.32)	1.21 (0.91–1.61)	0.99 (0.70–1.39)	1.11 (0.86–1.43)	1.13 (0.88–1.44)	0.98 (0.57–1.70)	0.79 (0.59–1.07)	0.93 (0.61–1.43)	1.0 (0.53–1.89)	0.97 (0.64–1.46)	0.77 (0.50–1.21)	1.50 (0.61–3.71)
No	Ref						Ref					
**Current alcohol use**												
Yes	**1.69 (1.31–2.20)**	**1.60 (1.07–2.38)**	**1.90 (1.25–2.88)**	1.24 (0.90–1.70)	**1.77 (1.26–2.49)**	**2.31 (1.23–4.33)**	1.29 (0.94–1.78)	1.35 (0.74–2.45)	1.34 (0.62–2.90)	1.37 (0.81–2.34)	0.99 (0.48–2.04)	1.26 (0.45–3.55)
No	Ref						Ref					
**Physical activity**												
Insufficient	**1.25 (1.05–1.49)**	1.03 (0.80–1.32)	1.22 (0.95–1.58)	1.13 (0.92–1.40)	1.19 (0.96–1.48)	0.97 (0.57–1.64)	**1.57 (1.27–1.93)**	1.42 (0.99–2.03)	0.91 (0.57–1.46)	0.81 (0.57–1.13)	1.38 (0.93–2.03)	0.73 (0.30–1.78)
Sufficient	Ref						Ref					
**Overweight including obesity**												
Yes	**1.94 (1.62–2.31)**	**2.18 (1.68–2.82)**	**2.07 (1.59–2.69)**	**2.74 (2.17–3.45)**	**2.11 (1.69–2.64)**	**1.93 (1.09–3.43)**	**1.61 (1.30–1.99)**	**2.89 (1.99–4.21)**	**2.39 (1.47–3.88)**	**2.10 (1.47–2.98)**	**2.30 (1.58–3.33)**	2.13 (0.87–5.21)
No	Ref						Ref					

Bold values represent statistical significance (*P* < 0.05)

**#** Owing to inadequate numbers for multivariate analysis, the reference category for Northeast was set as 18–49 years for raised fasting blood glucose

Adults between 50 and 69 years from the north (aOR: 16.74) and south parts of India (aOR 15.06) had the highest odds of risk for raised fasting blood glucose with least in the northeast (aOR: 2.97). Urban residents from the northeast (aOR: 2.26) and overweight/obese adults from the west (aOR: 2.89) were at increased risk for raised fasting blood glucose (Table 4)*.*

### Comparison of risk factors prevalence with HDI and its components across the regions

North and southern regions of India had high HDI scores of 0.7076 and 0.6955, respectively. These regions showed an increased prevalence of metabolic risk factors and clustering of at least 3 risk factors. Also, the ten-year CVD risk of ≥30% was highest in these regions when compared to the rest of India (Supplementary Table 6).

## Discussion

This study provides a snapshot view of the region-wise heterogeneity in the prevalence of risk factors for NCDs in India. There are significant gaps in the regional epidemiology of NCDs associated risk factors in India. To the best of our knowledge, this is the first attempt to analyse NCD risk factor data by region in India. We observed a predominance of behavioural risk factors (tobacco and alcohol use) in the northeast region and metabolic risk factors (overweight, obesity, raised blood pressure and raised fasting blood glucose) in the south. This predominance was similar in both sexes. These regional differences could be due to the rapid and different rates in urbanization, access and quality of health care, nutritious food and spaces for physical activity, economic stability, health sector preparedness, other behavioural, occupational, and environmental risks, etc. [1]

The current study findings show a high prevalence of smoking and smokeless tobacco use in the northeast. A study done by Lahoti et al. in 2021 showed a relative decrease in smoking by 5.7% but an increase in smokeless tobacco use (31.1%) between GATS-1 (2009–10) and GATS-2 (2016–17) in the northeast [29]. Shaikh et al. in 2022, reported that both smoking and smokeless tobacco use are most prevalent than the national average among men and women in north-eastern India (1998–2016 from NFHS) [30]. The current study results showed a high prevalence of insufficient physical activity in the north, east, central and south India (45–50%). The study in 2020 by Podder et al., reported that a higher proportion of people from the east (51.7%) met the WHO recommended levels of physical activity, whereas a lesser proportion from south India (28%) met these recommendations [31].

Our results revealed a high prevalence of obesity and raised fasting blood glucose in the south and a similar prevalence of raised blood pressure in the south and north. Chandrupatla et al. in 2020 analysis using the NFHS data showed a high prevalence of diabetes among the young and middle-aged adults from the southern region (9.39%) [32]. Mote BN in 2016, reported a high prevalence of hypertension among northern states followed by southern states [33].

Overall, a major proportion of adults from the south were exposed to all NCD risk factors when compared to other regions. Adults from the west showed low exposure to risk factors when compared to other regions except for the high prevalence of raised fasting blood glucose. In addition, both men and women from south India showed a high prevalence of clustering of ≥3 risk factors and 10-year CVD risk of ≥30% or with existing CVD. Contrasting to our study were the results from the cross-sectional analysis results from 2005 to 2016 NFHS data by Shaikh et al. in 2021 [34]. This study showed that men and women from the northeast showed a high prevalence and, the lowest prevalence of clustering of three or more lifestyle risk factors in the Southern region [34]. These differences between the studies can be attributed to the varied definitions of indicators, non-uniform methodology and age criteria. However, our findings are more representative of the clustering of risk factors as the study used standard global definitions of the risk factors and utilized the 2017–18 NNMS data [3, 14–16].

Regional analyses from the national sample have been periodically undertaken in various sectors to score economic development, communicable diseases, etc., at the national and international level to inform and facilitate actions [35–38]. Similar periodic approaches with uniform methods are useful for region-wise mapping of NCD risk factors in India like the National Health and Nutrition Evaluation Surveys in the United Kingdom [39]. Such monitoring mechanisms guide in strengthening and planning interventions to address NCD burden. The current study shows the need for strengthening efforts towards controlling metabolic risk factors in south India, both the behavioural and metabolic risk factors in the north, together with west and central parts of India, also reducing tobacco use in the northeast and encouraging physical activity in east India.

There is paucity of literature related to comprehensive NCD risk factor for different regions in India. This study provides an opportunity to fill the literature gaps and mark the discrepancies in the NCD risk factor profile across the six regions at a given point of time. It helps to set regional baseline for monitoring NCD indicators to be achieved by 2025. For subnational governments to implement all the actions needed to control and manage NCDs and their risk factors, they need comprehensive evidence on NCD risk factors. With the established methods, well-structured study tools, protocols, wide network of collaborators and regional evidence, they can set-up NCD surveillance systems.

The main strengths of the current study include the representativeness of sample, use of primary national survey data to arrive at the region-wise key behavioural (self-reported) and metabolic (both self–reported and measured) NCD risk factor estimates. The narrow confidence intervals among the risk factors indicates greater precision of the estimates. The robustness of these estimates can be attributed to the multistage sampling design, adequate sample power, standardised questionnaires (WHO-STEPS, and GATS, India) and show cards, rigorous training and quality control mechanisms, and high survey response rates. The study findings offer academicians and researchers to study these regional disparities and plan further research studies. Furthermore, regional estimates guide to frame culturally appropriate behaviour change communication strategies like encouraging self-care, and technological tools like mobile applications and health trackers, etc.

The current study has inadequate power to generate gender stratified estimates for rural versus urban subgroups, state and district specific NCD indicators. Future studies could be designed to arrive at both regional and national estimates. Since the study involves self-reported responses, there is a possibility of recall bias and under/over reporting of behavioural risk factors (tobacco, alcohol, physical activity and diet). However, adequate time was spent on each question ensuring participant privacy and confidentiality of responses.

## Conclusion

There are observed regional differences in the NCD risk factors among adults and the rapidly increasing burden of NCDs in India. These differences among risk factor prevalence have not been studied systematically and there is a need for sustainable monitoring mechanisms on risk factors most prevalent within the regions. This needs to be backed up by developing regional NCD action plans, standard methods and periodicity. These regional estimates from the present study provide the much-needed evidence to plan and target effective NCD control programs and facilitate the allocation of financial resources.

## Supplementary Information

**Additional file 1: Supplementary Table 1.** List of indicators, definitions and instruments used in the National NCD monitoring Survey (NNMS) 2017-18. **Supplementary Table 2.** Prevalence of behavioural risk factors associated with NCDs among adults aged 18-69 years in India by place of residence, sex, age group and region. **Supplementary Table 3.** Prevalence of metabolic risk factors associated with NCDs among adults aged 18-69 years in India by place of residence, sex, age group and region. **Supplementary Table 4.** Prevalence of clustering of ≥3 risk factors associated with NCDs among adults aged 18-69 years in India by place of residence, sex, age group and region. **Supplementary Table 5.** Prevalence of 30% or more 10 Year CVD risk or with existing CVD among adults aged 40-69 Years in India by place of residence, sex, age group and region. **Supplementary Table 6.** Comparison of prevalence of risk factors among adults in India with Human Development Index and its Components. **Supplementary Table 7.** Distribution of survey respondents for various risk factors by place of residence, sex, age group and region.

## Data Availability

The full report of NNMS is available at www.ncdirindia.org. Any further data are available upon reasonable request to the corresponding author.

## Abbreviations

NCDs: Noncommunicable Diseases
CVDs: Cardiovascular diseases
NNMS: National Noncommunicable Disease Monitoring Survey
CI: Confidence Interval
ICMR: Indian Council of Medical Research
NCDIR: National Centre for Disease Informatics and Research
PSUs: Primary Sampling Units
IDSP: Integrated Disease Surveillance Project
GATS: Global Adult Tobacco Survey
WHO: World Health Organization
MOHFW: Ministry of Health and Family Welfare
