# Supplementary material for: Regional estimates of noncommunicable diseases associated risk factors among adults in India: results from National Noncommunicable Disease Monitoring Survey
Source: BMC Public Health. 2022 May 30;22:1069. doi: 10.1186/s12889-022-13466-5 (PMC9150326; doi:10.1186/s12889-022-13466-5)
Supplement: Supplementary file 1 — Additional file 1: Supplementary Table 1. List of indicators, definitions and instruments used in the National NCD monitoring Survey (NNMS) 2017-18. Supplementary Table 2. Prevalence of behavioural risk factors associated with NCDs among adults aged 18-69 years in India by place of residence, sex, age group and region. Supplementary Table 3. Prevalence of metabolic risk factors associated with NCDs among adults aged 18-69 years in India by place of residence, sex, age group and region. Supplementary Table 4. Prevalence of clustering of ≥3 risk factors associated with NCDs among adults aged 18-69 years in India by place of residence, sex, age group and region. Supplementary Table 5. Prevalence of 30% or more 10 Year CVD risk or with existing CVD among adults aged 40-69 Years in India by place of residence, sex, age group and region. Supplementary Table 6. Comparison of prevalence of risk factors among adults in India with Human Development Index and its Components. Supplementary Table 7. Distribution of survey respondents for various risk factors by place of residence, sex, age group and region. [file 12889_2022_13466_MOESM1_ESM.docx]

**Supplementary table 1: List of indicators, definitions and instruments used in the National NCD monitoring Survey (NNMS) 2017-18.**

| **Indicator** | **Instruments** | **Definition** |
| --- | --- | --- |
| Current tobacco use | Questionnaire | Adults who used any form of tobacco (smoke and/or smokeless) in the last 12 months preceding the survey. |
| Current daily smoked tobacco use | Questionnaire | Adults who used any smoked tobacco products such as bidis, cigarettes, cigars, chillum, pipes, hookah or any other local smoked tobacco products daily in the last 12 months preceding the survey. |
| Current daily smokeless tobacco use | Questionnaire | Adults who used any smokeless tobacco products such as chewing tobacco, paan with tobacco, tobacco snuff by mouth or nose daily in the last 12 months preceding the survey. |
| Second hand tobacco smoke exposure at home | Questionnaire | Adults who reported being exposed to tobacco smoke on one or more than one occasion due to someone smoking tobacco close by at home in the past 30 days. |
| Current alcohol use | Questionnaire | Adults who consumed any form of alcohol (such as beer, wine, whisky, locally prepared alcohol, etc) in the last 12 months preceding the survey. |
| Heavy episodic drinking | Questionnaire | Adults those who engaged in consuming ≥6 standard drinks (equivalent to 60 grams of pure alcohol or ethanol) in a single drinking occasion over the past 30 days. |
| Insufficient physical activity | Questionnaire | Adults who engaged in <150 minutes of moderate – intensity physical activity per week OR <75 minutes of vigorous – intensity physical activity per week OR an equivalent combination of moderate- and vigorous- intensity physical activity accumulating <600 MET – minutes per week. |
| Overweight and obesity | Weight: Digital weighing scale (SECA 803) and Height: Portable stadiometer (SECA 213) | Overweight: BMI: 25.0–29.9 Kg/m^2^; Obesity: BMI≥ 30.0 Kg/m^2^ as per WHO cut-off. |
| Central Obesity | Waist circumference measured using Measuring or tension tape (SECA, 201) | Waist circumference of ≥90cm in males and ≥80cm in females (as per South Asia Pacific Guidelines). |
| Raised blood pressure | 1) Measurement of blood pressure using Automatic blood pressure machine (OMRON HEM–7120, Omron corporation, Kyoto, Japan). | Adults with a systolic blood pressure ≥140 mmHg and/or diastolic blood pressure ≥90 mmHg including those on medication for raised blood pressure. |
|  | 2) Questionnaire - Self reported medical history of raised blood pressure |  |
| Raised fasting blood glucose | 1) Fasting blood glucose using Glucometer (Gluco spark, Sensa core, Telangana, India) | Adults with fasting blood glucose value ≥126 mg/dl including those on medication for raised blood glucose. |
|  | 2) Questionnaire - Self reported medical history of raised blood glucose |  |
|  |  |  |

**Supplementary Table 2: Prevalence of behavioural risk factors associated with NCDs among adults aged 18-69 years in India by place of residence, sex, age group and region.**

|  | **Place of residence** | |  | **Sex** | | |  | | **Age group** | | | **Total** |
| --- | --- | --- | --- | --- | --- | --- | --- | --- | --- | --- | --- | --- |
| **Behavioural risk factor**s | **Urban** | **Rural** |  | **Men** | **Women** | |  | | **18-29 Years** | **30-49 Years** | **50-69 Years** |  |
|  | **% (95% CI)** | **% (95% CI)** |  | **% (95% CI)** | **% (95% CI)** | |  | | **% (95% CI)** | **% (95% CI)** | **% (95% CI)** | **% (95% CI)** |
| **Current tobacco use (smoke and/or smokeless variety)** (in last 12 months) | | | | | |  | |  | |  |  |  |
| South | 14.7 (13.0- 16.4) | 23.6 (21.3 – 26.0) |  | 32.5 (29.9 - 35.2) | 8.1 (6.8 - 9.5) | |  | | 12.6 (10.2 - 15.4) | 18.8 (16.7 - 20.9) | 21.8 (19.2 - 24.6) | 18.4 (17.0 - 19.9) |
| West | 27.3 (24.5 - 30.2) | 39.1 (35.4 – 43.0) |  | 49.2 (45.7 - 52.7) | 14.7 (12.3 - 17.3) | |  | | 21.7 (17.9 - 25.9) | 34.3 (31.0 - 37.9) | 38.0 (33.5 - 42.7) | 32.1 (29.8 - 34.4) |
| North | 21.3 (18.3 - 24.4) | 24.5 (21.4 – 28.0) |  | 44.1 (40.2 - 48.2) | 6.4 (4.8 - 8.3) | |  | | 17.1 (13.5 - 21.1) | 26.6 (23.2 - 30.2) | 22.5 (18.4 – 27.0) | 22.8 (20.7 - 25.1) |
| Central | 28.6 (25.6 - 31.7) | 40.2 (37.6 - 42.8) |  | 60.1 (57.1 – 63.0) | 14.0 (12.1 - 16.1) | |  | | 26.6 (23.4 - 30.1) | 38.5 (35.6 - 41.5) | 42.0 (37.9 - 46.3) | 35.8 (33.8 - 37.8) |
| East | 28.1 (25.1 - 31.3) | 34.2 (31.8 - 36.6) |  | 55.9 (52.8 – 59.0) | 13.5 (11.7 - 15.4) | |  | | 21.7 (18.7 – 25.0) | 34.4 (31.6 - 37.3) | 39.1 (35.2 - 43.1) | 32.0 (30.1 – 34.0) |
| Northeast | 48.8 (40.3 - 57.4) | 44.0 (38.0 - 50.2) |  | 61.8 (54.9 - 68.3) | 28.0 (21.9 - 34.9) | |  | | 32.5 (23.2 - 43.1) | 50.5 (43.4 - 57.7) | 47.4 (38.4 - 56.5) | 45.7 (40.7 - 50.7) |
| **Current daily smoked tobacco use**(in last 12 months) | | | | | | | | | | | | |
| South | 6.7 (5.6 – 8.0) | 8.3 (6.8 – 10.0) |  | 16.3 (14.3 - 18.5) | 0.8 (0.4 - 1.3) | |  | | 4.1 (2.7 - 5.8) | 7.1 (5.8 - 8.6) | 10.0 (8.2 - 12.1) | 7.4 (6.5 - 8.4) |
| West | 4.6 (3.4 - 6.1) | 3.3 (2.1 - 4.9) |  | 8.0 (6.3 - 10.1) | 0.0 ( - ) | |  | | 2.0 (0.9 - 3.7) | 3.9 (2.6 - 5.5) | 6.3 (4.3 – 9.0) | 4.1 (3.2 - 5.1) |
| North | 8.3 (6.4 - 10.5) | 11.8 (9.5 - 14.5) |  | 21.1 (17.9 - 24.5) | 1.4 (0.8 - 2.5) | |  | | 3.7 (2.1 - 5.9) | 11.0 (8.7 - 13.7) | 14.9 (11.5 - 18.9) | 10.0 (8.5 - 11.7) |
| Central | 8.2 (6.5 - 10.2) | 12.6 (10.9 - 14.4) |  | 21.6 (19.2 - 24.2) | 1.4 (0.8 - 2.1) | |  | | 4.9 (3.5 - 6.7) | 11.7 (9.8 - 13.7) | 17.1 (14.1 - 20.4) | 10.9 (9.7 - 12.3) |
| East | 8.2 (6.5 - 10.3) | 7.7 (6.4 - 9.1) |  | 17.3 (15.1 - 19.8) | 0.5 (0.2 - 1.1) | |  | | 5.0 (3.5 - 6.9) | 7.4 (5.9 – 9.0) | 12.0 (9.6 - 14.9) | 7.9 (6.8 – 9.0) |
| Northeast | 21.7 (15.3 - 29.4) | 12.3 (8.7 - 16.8) |  | 24.6 (19.0 - 30.9) | 5.5 (2.9 - 9.5) | |  | | 7.2 (3.1 - 14.3) | 19.0 (13.9 - 25.2) | 15.8 (10.0 - 23.3) | 15.5 (12.1 - 19.4) |
| **Current daily smokeless tobacco use** (in last 12 months) | | | | | | | | | | | | |
| South | 6.0 (4.9 - 7.2) | 13.2 (11.4 - 15.2) |  | 12.4 (10.7 - 14.4) | 6.6 (5.5 - 7.8) | |  | | 6.2 (4.5 - 8.3) | 8.8 (7.4 - 10.5) | 11.3 (9.4 - 13.5) | 9.1 (8.0 - 10.2) |
| West | 17.8 (15.4 - 20.4) | 29.5 (26.0 - 33.1) |  | 32.7 (29.5 – 36.0) | 12.2 (10.1 - 14.7) | |  | | 14.5 (11.3 - 18.2) | 25.4 (22.3 - 28.6) | 25.4 (21.4 - 29.6) | 22.6 (20.5 - 24.7) |
| North | 11.0 (8.8 - 13.5) | 11.0 (8.8 - 13.6) |  | 20.2 (17.1 - 23.6) | 3.9 (2.7 - 5.5) | |  | | 10.0 (7.3 - 13.3) | 13.6 (11.1 - 16.5) | 7.6 (5.2 - 10.7) | 11.0 (9.4 - 12.8) |
| Central | 18.0 (15.5 - 20.7) | 27.6 (25.3 – 30.0) |  | 38.2 (35.4 - 41.2) | 11.2 (9.5 – 13.0) | |  | | 19.2 (16.4 - 22.3) | 25.8 (23.2 - 28.6) | 26.3 (22.7 - 30.1) | 24.0 (22.2 - 25.8) |
| East | 19.7 (17.1 - 22.6) | 23.0 (20.9 - 25.2) |  | 36.2 (33.2 - 39.2) | 10.7 (9.1 - 12.4) | |  | | 13.0 (10.5 - 15.7) | 24.3 (21.8 – 27.0) | 27.0 (23.5 - 30.7) | 21.8 (20.2 - 23.6) |
| Northeast | 31.8 (24.2 - 40.2) | 34.5 (28.9 - 40.5) |  | 42.7 (36.0 - 49.7) | 23.6 (17.9 - 30.2) | |  | | 20.5 (12.9 - 30.1) | 39.7 (32.8 - 46.9) | 33.3 (25.2 - 42.3) | 33.6 (29.0 - 38.4) |
| **Second hand tobacco smoke exposure at home** (in past 30 days) | | | | | | | | | | | | |
| South | 18.7 (16.8 - 20.6) | 21.8 (19.5 - 24.2) |  | 23.0 (20.7 - 25.4) | 17.8 (16.0 - 19.7) | |  | | 22.7 (19.5 - 26.1) | 20.5 (18.4 - 22.8) | 17.4 (15.0 - 19.9) | 20.0 (18.5 - 21.5) |
| West | 25.8 (23.1 - 28.7) | 37.9 (34.2 - 41.7) |  | 34.1 (30.8 - 37.5) | 27.3 (24.2 - 30.5) | |  | | 29.4 (25.1 – 34.0) | 33.1 (29.8 - 36.6) | 27.9 (23.8 - 32.3) | 30.7 (28.5 - 33.1) |
| North | 30.7 (27.3 - 34.2) | 44.3 (40.5 - 48.2) |  | 41.3 (37.3 - 45.3) | 34.2 (30.9 - 37.6) | |  | | 39.6 (34.8 - 44.6) | 37.5 (33.7 - 41.4) | 34.3 (29.5 - 39.3) | 37.3 (34.7 - 39.9) |
| Central | 26.7 (23.8 - 29.7) | 35.1 (32.6 - 37.6) |  | 41.3 (38.3 - 44.3) | 23.5 (21.2 – 26.0) | |  | | 33.9 (30.4 - 37.6) | 31.6 (28.9 - 34.5) | 29.8 (26.1 - 33.8) | 31.9 (30.0 - 33.8) |
| East | 37.2 (33.9 - 40.6) | 31.1 (28.7 - 33.5) |  | 36.1 (33.1 - 39.1) | 31.0 (28.5 - 33.6) | |  | | 31.6 (28.1 - 35.2) | 33.8 (31.0 - 36.7) | 33.9 (30.1 - 37.8) | 33.2 (31.3 - 35.2) |
| Northeast | 42.6 (34.3 - 51.3) | 38.9 (33.0 – 45.0) |  | 37.7 (31.2 - 44.6) | 42.9 (35.8 - 50.1) | |  | | 49.4 (38.8 – 60.0) | 37.5 (30.7 - 44.6) | 37.7 (29.2 - 46.8) | 40.2 (35.3 - 45.1) |
| **Current alcohol use** (in last 12 months) | | | | | | | | | | | | |
| South | 15.3 (13.6 - 17.1) | 16.9 (14.8 - 19.1) |  | 34.7 (32.0 - 37.4) | 2.2 (1.6 – 3.0) | |  | | 13.9 (11.4 - 16.8) | 17.7 (15.7 - 19.8) | 14.8 (12.6 - 17.2) | 15.9 (14.6 - 17.3) |
| West | 9.6 (7.8 - 11.6) | 10.8 (8.5 - 13.4) |  | 19.2 (16.5 – 22.0) | 0.8 (0.3 - 1.6) | |  | | 5.7 (3.8 - 8.3) | 12.0 (9.8 - 14.5) | 10.8 (8.1 – 14.0) | 10.1 (8.6 - 11.6) |
| North | 12.3 (10.0 - 14.9) | 10.4 (8.3 – 13.0) |  | 25.5 (22.1 - 29.1) | 0.5 (0.2 - 1.2) | |  | | 7.9 (5.5 - 10.9) | 14.0 (11.4 - 16.9) | 10.7 (7.8 - 14.2) | 11.4 (9.8 - 13.2) |
| Central | 15.6 (13.3 - 18.2) | 14.4 (12.6 - 16.3) |  | 30.8 (28.1 - 33.6) | 0.6 (0.3 - 1.2) | |  | | 14.1 (11.7 - 16.9) | 17.0 (14.8 - 19.3) | 11.6 (9.1 - 14.6) | 14.9 (13.4 - 16.4) |
| East | 7.5 (5.8 - 9.5) | 11.3 (9.8 – 13.0) |  | 20.1 (17.7 - 22.7) | 2.1 (1.4 – 3.0) | |  | | 9.7 (7.6 - 12.2) | 11.1 (9.3 - 13.1) | 8.3 (6.2 - 10.7) | 10.0 (8.8 - 11.3) |
| Northeast | 28.7 (21.4 - 36.9) | 19.0 (14.6 - 24.2) |  | 35.2 (28.8 – 42.0) | 8.2 (4.9 - 12.9) | |  | | 19.3 (11.9 - 28.7) | 28.3 (22.1 - 35.1) | 14.9 (9.3 - 22.3) | 22.3 (18.3 - 26.7) |
| **Heavy episodic drinking^1^** | | | | | | | | | | | | |
| South | 6.0(4.9 - 7.2) | 5.8 (4.6 - 7.2) |  | 13.8 (12.0 - 15.9) | 0.1 (0.0- 0.3) | |  | | 3.9 (2.6 - 5.6) | 7.6 (6.3 - 9.2) | 4.8 (3.5 - 6.3) | 5.9 (5.1 - 6.8) |
| West | 3.3 (2.3 - 4.6) | 4.3 (2.9 - 6.1) |  | 7.2 (5.5 - 9.1) | 0.1 (0.0 - 0.6) | |  | | 0.7 (0.2 – 2.0) | 5.2 (3.8 – 7.0) | 3.8 (2.3 - 5.9) | 3.7 (2.8 - 4.7) |
| North | 5.6 (4.0 - 7.4) | 4.3 (2.9 - 6.1) |  | 11.4 (9.0 - 14.1) | 0.0 ( - ) | |  | | 3.1 (1.7 - 5.3) | 6.3 (4.6 - 8.5) | 4.5 (2.7 – 7.0) | 5.0 (3.9 - 6.2) |
| Central | 5.8 (4.3 - 7.5) | 5.9 (4.7 - 7.2) |  | 12.2 (10.3 - 14.3) | 0.2 (0.0 - 0.5) | |  | | 4.8 (3.3 - 6.6) | 7.3 (5.9 – 9.0) | 4.3 (2.8 - 6.3) | 5.8 (4.9 - 6.9) |
| East | 2.9 (1.9 - 4.2) | 3.3 (2.5 - 4.3) |  | 6.1 (4.8 - 7.7) | 0.9 (0.5 - 1.5) | |  | | 3.1 (2.0 - 4.7) | 3.4 (2.4 - 4.6) | 2.8 (1.6 - 4.3) | 3.2 (2.5 - 3.9) |
| Northeast | 9.3 (5.2 - 15.2) | 6.3 (3.8 - 9.9) |  | 13.6 (9.3 - 18.8) | 0.5 (0.1 - 2.5) | |  | | 2.4 (0.5 - 7.5) | 10.9 (7.0 – 16.0) | 5.3 (2.2 - 10.5) | 7.3 (5.0 - 10.3) |
| **Insufficient physical activity^2^** | | | | | | | | | | | | |
| South | 49.8 (47.3 - 52.2) | 37.5 (34.8 - 40.3) |  | 35.7 (33.0 - 38.5) | 50.9 (48.5 - 53.4) | |  | | 43.6 (39.7 - 47.6) | 42.0 (39.3 - 44.7) | 48.9 (45.7 - 52.1) | 44.6 (42.7 - 46.4) |
| West | 44.6 (41.4 - 47.9) | 30.9 (27.3 - 34.6) |  | 34.8 (31.5 - 38.2) | 43.3 (39.8 - 46.9) | |  | | 34.3 (29.7 - 39.1) | 38.2 (34.7 - 41.8) | 45.0 (40.2 - 49.8) | 39.1 (36.6 - 41.5) |
| North | 58.2 (54.5 - 61.8) | 40.2 (36.5 – 44.0) |  | 42.2 (38.2 - 46.2) | 55.2 (51.7 - 58.7) | |  | | 46.1 (41.1 - 51.1) | 45.6 (41.7 - 49.6) | 60.1 (55.0 - 65.1) | 49.6 (46.9 - 52.2) |
| Central | 59.9 (56.6 - 63.2) | 39.4 (36.8 – 42.0) |  | 38.6 (35.7 - 41.6) | 54.7 (51.9 - 57.6) | |  | | 46.3 (42.5 - 50.1) | 43.8 (40.8 - 46.9) | 54.8 (50.5 – 59.0) | 47.2 (45.1 - 49.3) |
| East | 56.5 (53.0 - 59.9) | 44.6 (42.1 - 47.2) |  | 26.7 (24.0 - 29.5) | 65.7 (63.0 - 68.2) | |  | | 49.2 (45.3 - 53.1) | 46.1 (43.1 - 49.2) | 53.1 (49.0 - 57.1) | 48.8 (46.7 - 50.8) |
| Northeast | 43.7 (35.2 - 52.4) | 33.3 (27.7 - 39.3) |  | 22.3 (16.9 - 28.5) | 52.5 (45.2 - 59.7) | |  | | 37.8 (27.9 - 48.6) | 32.4 (25.9 - 39.5) | 43.0(34.2 - 52.2) | 36.8 (32.0 - 41.7) |
|  |  |  |  |  |  | |  | |  |  |  |  |

1. Heavy episodic drinking constitutes those who reported drinking ≥6 standard drinks (equivalent to 60 grams of pure alcohol or ethanol) in a single drinking occasion in last 30 days of interview.
2. Insufficient physical activity constitutes those who engaged in <150 minutes of moderate – intensity physical activity per week OR <75 minutes of vigorous – intensity physical activity per week OR an equivalent combination of moderate- and vigorous- intensity physical activity accumulating <600 MET – minutes per week.

**Supplementary Table 3: Prevalence of metabolic risk factors associated with NCDs among adults aged 18-69 years in India by place of residence, sex, age group and region.**

| **Metabolic risk factor**s | **Place of residence** | |  | **Sex** | | |  | | **Age group** | | | | | | **Total** | |
| --- | --- | --- | --- | --- | --- | --- | --- | --- | --- | --- | --- | --- | --- | --- | --- | --- |
|  | **Urban** | **Rural** |  | **Men** | **Women** | |  | | **18-29 Years** | | **30-49 Years** | | **50-69 Years** | |  | |
|  | **% (95% CI)** | **% (95% CI)** |  | **% (95% CI)** | **% (95% CI)** | |  | | **% (95% CI)** | | **% (95% CI)** | | **% (95% CI)** | | **% (95% CI)** | |
| **Overweight (BMI ≥ 25.0 Kg/m^2^)** | | | | | | | | | | | | | | | | |
| South | 50.3 (47.8 - 52.7) | 28.8 (26.3 - 31.4) |  | 36.2 (33.5 - 38.9) | 44.7 (42.3 - 47.2) | |  | | 28.8 (25.3 - 32.6) | | 45.5 (42.8 - 48.2) | | 43.0 (39.8 - 46.3) | | 41.1 (39.3 - 42.9) | |
| West | 43.0 (39.8 - 46.3) | 20.4 (17.3 - 23.7) |  | 28.6 (25.5 - 31.9) | 39.1 (35.6 - 42.7) | |  | | 18.9 (15.2 - 23.1) | | 38.9 (35.3 - 42.5) | | 38.3 (33.7 - 43.1) | | 33.7 (31.3 - 36.1) | |
| North | 46.5 (42.8 - 50.3) | 26.6 (23.3 - 30.2) |  | 29.8 (26.1 - 33.6) | 42.4 (38.8 – 46.0) | |  | | 21.2 (17.2 - 25.6) | | 40.2 (36.3 - 44.2) | | 47.7 (42.5 – 53.0) | | 36.9 (34.3 - 39.5) | |
| Central | 37.5 (34.3 - 40.8) | 13.8 (12.1 - 15.7) |  | 20.0 (17.7 - 22.5) | 25.4 (23.0 – 28.0) | |  | | 12.7 (10.3 - 15.5) | | 27.2 (24.5 – 30.0) | | 26.7 (23.0 - 30.5) | | 22.8 (21.1 - 24.6) | |
| East | 35.4 (32.1 - 38.8) | 18.6 (16.7 - 20.7) |  | 20.0 (17.5 - 22.5) | 28.1 (25.6 - 30.6) | |  | | 17.6 (14.8 - 20.8) | | 28.9 (26.2 - 31.8) | | 23.6 (20.3 - 27.2) | | 24.5 (22.7 - 26.3) | |
| Northeast | 36.2 (28.2 - 44.8) | 15.3 (11.3 - 20.2) |  | 20.6 (15.4 - 26.6) | 24.4 (18.5 - 31.2) | |  | | 12.5 (6.6 – 21.0) | | 27.6 (21.5 - 34.5) | | 21.1 (14.4 - 29.2) | | 22.4 (18.4 - 26.8) | |
| **Obesity (BMI ≥30.0 Kg/m^2^)** | | | | | | | | | | | | | | | |  |
| South | 16.4 (14.7 - 18.3) | 7.1 (5.8 - 8.7) |  | 9.0 (7.4 - 10.7) | 15.0 (13.3 - 16.8) | |  | | 7.8 (5.9 - 10.2) | | 13.8 (12.0 - 15.8) | | 13.6 (11.5 - 15.9) | | 12.5 (11.3 - 13.7) | |
| West | 12.6 (10.5 - 14.9) | 5.2 (3.7 - 7.2) |  | 5.9 (4.4 - 7.7) | 13.4 (11.1 – 16.0) | |  | | 3.5 (2.0 - 5.7) | | 11.0 (8.8 - 13.4) | | 12.7 (9.7 - 16.2) | | 9.5 (8.1 - 11.1) | |
| North | 13.8 (11.4 - 16.6) | 8.1 (6.1 - 10.4) |  | 6.5 (4.7 - 8.8) | 14.5 (12.1 - 17.2) | |  | | 5.5 (3.5 - 8.2) | | 11.2 (8.9 – 14.0) | | 16.5 (12.9 - 20.7) | | 11.0 (9.4 - 12.8) | |
| Central | 10.6 (8.6 - 12.8) | 2.6 (1.8 - 3.5) |  | 3.8 (2.7 – 5.0) | 7.3 (5.9 – 9.0) | |  | | 1.7 (0.9 – 3.0) | | 8.1 (6.6 - 9.9) | | 5.5 (3.8 - 7.7) | | 5.6 (4.7 - 6.7) | |
| East | 9.1 (7.2 - 11.3) | 2.6 (1.9 - 3.5) |  | 3.3 (2.3 - 4.5) | 6.1 (4.9 - 7.6) | |  | | 3.1 (2.0 - 4.7) | | 6.1 (4.7 - 7.6) | | 4.5 (3.1 - 6.5) | | 4.9 (4.0 - 5.8) | |
| Northeast | 5.5 (2.5 - 10.5) | 2.8 (1.3 - 5.5) |  | 2.5 (1.0 - 5.4) | 5.1 (2.6 - 9.1) | |  | | 1.3 (0.1 - 5.7) | | 4.4 (2.1 - 8.2) | | 4.4 (1.7 - 9.3) | | 3.7 (2.2 – 6.0) | |
| **Central obesity^1^** | | | | | | | | | | | | | | | |  |
| South | 57.8 (55.4 - 60.2) | 38.4 (35.6 - 41.2) |  | 38.3 (35.6 - 41.1) | 57.7 (55.3 - 60.1) | |  | | 30.0 (26.4 - 33.7) | | 53.3 (50.5 – 56.0) | | 57.2 (53.9 - 60.4) | | 49.5 (47.7 - 51.4) | |
| West | 44.3 (41.0 - 47.6) | 21.2 (18.1 - 24.5) |  | 30.4 (27.2 - 33.7) | 39.4 (35.9 – 43.0) | |  | | 14.4 (11.1 - 18.2) | | 39.4 (35.8 - 43.1) | | 45.4 (40.6 - 50.2) | | 34.7 (32.4 - 37.2) | |
| North | 59.9 (56.1 - 63.5) | 35.5 (31.8 - 39.3) |  | 35.6 (31.7 - 39.5) | 57.7 (54.1 - 61.3) | |  | | 26.0 (21.7 - 30.7) | | 51.0 (47.0 – 55.0) | | 66.0 (60.9 - 70.8) | | 48.0 (45.3 - 50.7) | |
| Central | 43.8 (40.4 - 47.1) | 22.3 (20.1 - 24.5) |  | 23.7 (21.2 - 26.3) | 36.6 (33.8 - 39.4) | |  | | 13.5 (11.0 - 16.3) | | 35.9 (33.0 - 38.9) | | 40.2 (36.1 - 44.4) | | 30.4 (28.5 - 32.4) | |
| East | 44.1 (40.6 - 47.6) | 24.8 (22.6 – 27.0) |  | 16.9 (14.6 - 19.3) | 43.1 (40.4 - 45.9) | |  | | 19.4 (16.4 - 22.7) | | 35.6 (32.8 - 38.6) | | 36.8 (32.9 - 40.8) | | 31.5 (29.6 - 33.4) | |
| Northeast | 41.7 (33.4 - 50.4) | 22.5 (17.6 – 28.0) |  | 16.6 (11.9 - 22.2) | 42.9 (35.8 - 50.3) | |  | | 17.5 (10.4 - 26.9) | | 32.4 (25.9 - 39.5) | | 31.6 (23.6 - 40.5) | | 29.0 (24.6 - 33.7) | |
| **Raised blood pressure^2^** | | | | | | | | | | | | | | | |  |
| South | 37.6 (35.3 – 40.0) | 32.9 (30.3 - 35.6) |  | 36.5 (33.8 - 39.3) | 35.0 (32.7 - 37.3) | |  | | 12.0 (9.6 - 14.8) | | 30.8 (28.3 - 33.3) | | 58.3 (55.1 - 61.5) | | 35.6 (33.9 - 37.4) | |
| West | 29.8 (26.9 - 32.9) | 26.0 (22.7 - 29.5) |  | 28.8 (25.7 – 32.0) | 27.8 (24.7 – 31.0) | |  | | 10.8 (8.1 - 14.2) | | 27.1 (23.9 - 30.4) | | 46.7 (42.0 - 51.5) | | 28.3 (26.1 - 30.6) | |
| North | 37.4 (33.8 – 41.0) | 32.9 (29.3 - 36.6) |  | 39.5 (35.6 - 43.5) | 31.9 (28.7 - 35.3) | |  | | 17.8 (14.2 - 21.9) | | 32.6 (28.9 - 36.4) | | 58.1 (53.0 - 63.2) | | 35.2 (32.7 - 37.8) | |
| Central | 33.8 (30.7 – 37.0) | 21.8 (19.6 – 24.0) |  | 27.0 (24.3 - 29.7) | 25.8 (23.3 - 28.3) | |  | | 11.6 (9.3 - 14.2) | | 26.8 (24.1 - 29.5) | | 43.9 (39.7 - 48.1) | | 26.3 (24.5 - 28.2) | |
| East | 31.6 (28.4 - 34.9) | 25.2 (23.1 - 27.5) |  | 26.5 (23.8 - 29.3) | 28.2 (25.8 - 30.7) | |  | | 13.7 (11.2 - 16.5) | | 25.6 (23.0 - 28.3) | | 45.9 (41.8 - 49.9) | | 27.4 (25.6 - 29.3) | |
| Northeast | 34.9 (27.1 - 43.4) | 28.7 (23.4 - 34.5) |  | 35.2 (28.8 – 42.0) | 26.0 (20.0 - 32.7) | |  | | 9.6 (4.7 - 17.4) | | 27.9 (21.8 - 34.7) | | 50.9 (41.8 - 59.9) | | 30.8 (26.3 - 35.6) | |
| **Raised fasting blood glucose^3^** | | | | | |  | |  | |  | |  | |  | |  |
| South | 24.8 (22.6 - 27.1) | 16.6 (14.5 - 18.9) |  | 20.5 (18.2 – 23.0) | 21.8 (19.7 - 23.9) | |  | | 3.7 (2.3 - 5.6) | | 17.3 (15.2 - 19.5) | | 37.8 (34.6 - 41.1) | | 21.2 (19.7 - 22.8) | |
| West | 14.8 (12.4 - 17.4) | 7.5 (5.5 - 9.9) |  | 11.5 (9.2 – 14.0) | 12.1 (9.8 - 14.8) | |  | | 2.9 (1.5 – 5.0) | | 10.1 (8.0 - 12.7) | | 22.9 (18.9 - 27.4) | | 11.8 (10.1 - 13.6) | |
| North | 10.9 (8.6 - 13.5) | 5.4 (3.8 - 7.5) |  | 8.1 (6.0 - 10.7) | 8.2 (6.3 - 10.5) | |  | | 1.2 (0.4 - 2.8) | | 5.0 (3.4 – 7.0) | | 21.0 (16.8 - 25.7) | | 8.2 (6.7 - 9.8) | |
| Central | 11.6 (9.4 – 14.0) | 6.3 (5.1 - 7.8) |  | 7.5 (6.0 - 9.3) | 9.0 (7.4 - 10.8) | |  | | 2.6 (1.6 - 4.1) | | 7.4 (5.9 - 9.2) | | 16.6 (13.6 – 20.0) | | 8.3 (7.2 - 9.5) | |
| East | 10.5 (8.4 – 13.0) | 5.2 (4.2 - 6.5) |  | 6.0 (4.6 - 7.7) | 7.8 (6.4 - 9.5) | |  | | 1.8 (0.9 - 3.1) | | 6.3 (4.9 – 8.0) | | 13.8 (11.1 - 16.9) | | 7.0 (6.0 - 8.2) | |
| Northeast | 14.9 (8.9 - 22.7) | 6.6 (3.9 - 10.4) |  | 10.8 (6.8 - 16.1) | 7.5 (4.1 - 12.3) | |  | | 1.5 (0.2 - 6.9) | | 8.8 (5.1 – 14.0) | | 14.6 (8.8 - 22.3) | | 9.1 (6.4 - 12.6) | |
|  |  |  |  |  |  | |  | |  | |  | |  | |  | |

1. Central obesity was defined as having waist circumference of ≥90 cm in males and ≥80 cm in females.
2. Raised blood pressure was when the systolic blood pressure ≥140 mm of Hg and/or diastolic blood pressure ≥90 mm of Hg including those on medication for raised BP among adults aged 18-69 years.
3. Raised fasting blood glucose were when the values of fasting blood glucose were ≥126 mg/dl including those on medication for raised blood glucose among adults aged 18-69 years.

**Supplementary Table 4: Prevalence of clustering of ≥3 risk factors associated with NCDs among adults aged 18-69 years in India by place of residence, sex, age group and region.**

| **Clustering of ≥3 risk factors** | **Place of residence** | |  | **Sex** | |  | **Age group** | | | **Total** |
| --- | --- | --- | --- | --- | --- | --- | --- | --- | --- | --- |
|  | **Urban** | **Rural** |  | **Men** | **Women** |  | **18-29 Years** | **30-49 Years** | **50-69 Years** |  |
|  | **% (95% CI)** | **% (95% CI)** |  | **% (95% CI)** | **% (95% CI)** |  | **% (95% CI)** | **% (95% CI)** | **% (95% CI)** | **% (95% CI)** |
| South | 56.2 (53.6 - 58.8) | 42.2 (39.3 - 45.1) |  | 50.4 (47.4 - 53.4) | 49.9 (47.3 - 52.4) |  | 21.9 (18.6 - 25.6) | 50.6 (47.8 - 53.5) | 67.0 (63.8 - 70.1) | 50.1 (48.2 – 52.0) |
| West | 49.0 (45.5 - 52.5) | 35.3 (31.4 - 39.4) |  | 47.2 (43.4 – 51.0) | 39.3 (35.6 - 43.2) |  | 19.3 (15.3 - 23.8) | 44.8 (40.8 - 48.7) | 62.7 (57.7 - 67.5) | 43.4 (40.7 - 46.1) |
| North | 53.3 (49.3 - 57.2) | 39.2 (35.3 - 43.3) |  | 49.0 (44.8 - 53.3) | 44.2 (40.5 - 48.1) |  | 25.5 (21.0 - 30.5) | 47.5 (43.3 - 51.7) | 66.0 (60.7 - 71.1) | 46.4 (43.5 - 49.2) |
| Central | 54.0 (50.5 - 57.6) | 32.9 (30.4 - 35.5) |  | 44.1 (41.0 - 47.3) | 37.7 (34.9 - 40.7) |  | 22.9 (19.6 - 26.4) | 41.9 (38.8 - 45.1) | 58.9 (54.6 - 63.2) | 40.8 (38.6 - 42.9) |
| East | 49.3 (45.5 – 53.0) | 34.8 (32.3 - 37.4) |  | 37.8 (34.7 – 41.0) | 41.2 (38.3 - 44.1) |  | 22.2 (18.9 - 25.8) | 40.6 (37.5 - 43.7) | 56.1 (51.8 - 60.2) | 39.7 (37.6 - 41.8) |
| Northeast | 57.4 (47.7 - 66.7) | 37.4 (31.2 - 43.9) |  | 42.5 (35.2 - 50.1) | 44.9 (37.2 - 52.7) |  | 20.3 (11.9 - 31.3) | 45.5 (37.8 - 53.3) | 55.3 (45.7 - 64.7) | 43.7 (38.3 - 49.1) |
| ***p value*** | ***0.007*** | ***<0.001*** |  | ***<0.001*** | ***<0.001*** |  | ***0.545*** | ***<0.001*** | ***<0.001*** | ***<0.001*** |
|  |  |  |  |  |  |  |  |  |  |  |

**Supplementary Table 5: Prevalence of 30% or more 10 Year CVD risk or with existing CVD among adults aged 40-69 Years in India by place of residence, sex, age group and region.**

| **Ten Year CVD risk** | **Place of residence** | |  | **Sex** | |  | **Age group** | | |  | **Total** |
| --- | --- | --- | --- | --- | --- | --- | --- | --- | --- | --- | --- |
|  | **Urban** | **Rural** |  | **Men** | **Women** |  | **40-49 Years** | **50-59 Years** | **60-69 Years** |  |  |
|  | **% (95% CI)** | **% (95% CI)** |  | **% (95% CI)** | **% (95% CI)** |  | **% (95% CI)** | **% (95% CI)** | **% (95% CI)** |  | **% (95% CI)** |
| South | 17.1 (14.2 - 20.2) | 19.5 (16.0 - 23.5) |  | 19.9 (16.3 - 23.8) | 16.8 (13.9 – 20.0) |  | 12.1 (9.0 - 15.7) | 14.8 (11.4 - 18.9) | 28.6 (23.8 - 33.7) |  | 18.1 (15.8 - 20.5) |
| West | 14.5 (10.6 - 19.2) | 11.4 (7.4 - 16.8) |  | 14.2 (10.2 - 19.2) | 12.1 (8.1 - 17.2) |  | 5.6 (2.8 - 9.9) | 16.5 (11.0 - 23.5) | 19.3 (13.3 - 26.5) |  | 13.3 (10.3 - 16.7) |
| North | 18.1 (13.5 - 23.6) | 13.3 (8.8 - 19.1) |  | 20.3 (15.0 - 26.6) | 12.3 (8.3 - 17.3) |  | 8.3 (4.6 - 13.6) | 16.3 (10.7 - 23.4) | 25.9 (18.5 - 34.5) |  | 16.1 (12.7 – 20.0) |
| Central | 9.7 (6.6 - 13.7) | 10.1 (7.2 - 13.7) |  | 14.0 (10.5 - 18.3) | 5.7 (3.5 - 8.8) |  | 4.6 (2.5 - 7.9) | 9.4 (5.9 - 14.1) | 18.2 (12.9 - 24.6) |  | 9.9 (7.7 - 12.5) |
| East | 12.2 (8.6 - 16.6) | 6.8 (4.8 - 9.4) |  | 8.6 (6.0 – 12.0) | 8.8 (6.3 – 12.0) |  | 3.3 (1.7 - 5.8) | 7.0 (4.1 – 11.0) | 18.9 (13.9 - 24.8) |  | 8.7 (6.8 – 11.0) |
| Northeast | 17.1 (7.5 – 32.0) | 13.7 (7.3 - 22.9) |  | 12.9 (6.3 - 22.9) | 17.4 (8.6 - 30.2) |  | 2.8 (0.3 - 12.3) | 10.7 (3.1 - 25.9) | 27.3 (15.9 - 41.6) |  | 14.8 (9.1 - 22.4) |
| ***p value*** | ***0.046*** | ***<0.001*** |  | ***<0.001*** | ***<0.001*** |  | ***<0.001*** | ***0.022*** | ***0.042*** |  | ***<0.001*** |
|  |  |  |  |  |  |  |  |  |  |  |  |

Abbreviations: CVD: Cardiovascular diseases

**Supplementary Table 6: Comparison of prevalence of risk factors among adults in India with Human Development Index and its Components**

| **Region** | **Behavioural risk factors** | | | **Metabolic risk factors** | | | | **Clustering of >=3 risk factors** | **>=30% or with existing CVD risk (40-69 Years)** |  | **HDI and its components (2017-18)** | | | |
| --- | --- | --- | --- | --- | --- | --- | --- | --- | --- | --- | --- | --- | --- | --- |
|  | **Current Tobacco use** | **Current Alcohol use** | **Insufficient physical activity** | **Overweight including obesity** | **Central obesity** | **Raised blood pressure** | **Raised fasting blood glucose** |  |  |  | **HDI Value** | **Health Index** | **Income Index** | **Education Index** |
| **South** | 18.4 | 15.9 | 44.6 | 41.1 | 49.5 | 35.6 | 21.2 | 50.1 | 18.1 |  | 0.6955 | 0.8048 | 0.6870 | 0.6094 |
| **West** | 32.1 | 10.1 | 39.1 | 33.7 | 34.7 | 28.3 | 11.8 | 43.4 | 13.3 |  | 0.6820 | 0.7905 | 0.6865 | 0.5860 |
| **North** | 22.8 | 11.4 | 49.6 | 36.9 | 48.0 | 35.2 | 8.2 | 46.4 | 16.1 |  | 0.7076 | 0.7860 | 0.7219 | 0.6256 |
| **Central** | 35.8 | 14.9 | 47.2 | 22.8 | 30.4 | 26.3 | 8.3 | 40.8 | 9.9 |  | 0.6032 | 0.7178 | 0.5870 | 0.5210 |
| **East** | 32.0 | 10.0 | 48.8 | 24.5 | 31.5 | 27.4 | 7.0 | 43.7 | 14.8 |  | 0.6034 | 0.7581 | 0.5609 | 0.5170 |
| **Northeast** | 45.7 | 22.3 | 36.8 | 22.4 | 29.0 | 30.8 | 9.1 | 43.7 | 14.8 |  | 0.6799 | 0.7840 | 0.6549 | 0.6142 |

Abbreviations: CVD= Cardiovascular Diseases; HDI=Human Development Index

**Supplementary Table 7: Distribution of survey respondents for various risk factors by place of residence, sex, age group and region.**

| **Risk factors** | **Place of residence** | |  | **Sex** | |  | **Age group** | | | **Total** |
| --- | --- | --- | --- | --- | --- | --- | --- | --- | --- | --- |
|  | **Urban** | **Rural** |  | **Men** | **Women** |  | **18-29 Years** | **30-49 Years** | **50-69 Years** |  |
|  | **N** | **N** |  | **N** | **N** |  | **N** | **N** | **N** |  |
| **Tobacco and alcohol use** | | |  |  |  |  |  |  |  |  |
| South | 1645 | 1204 |  | 1206 | 1643 |  | 617 | 1311 | 921 | 2849 |
| West | 921 | 631 |  | 783 | 769 |  | 401 | 725 | 426 | 1552 |
| North | 701 | 652 |  | 589 | 764 |  | 381 | 616 | 356 | 1353 |
| Central | 851 | 1391 |  | 1059 | 1183 |  | 672 | 1037 | 533 | 2242 |
| East | 801 | 1481 |  | 998 | 1284 |  | 640 | 1061 | 581 | 2282 |
| Northeast | 129 | 252 |  | 199 | 182 |  | 83 | 184 | 114 | 381 |
| Total | 5048 | 5611 |  | 4834 | 5825 |  | 2794 | 4934 | 2931 | 10659 |
| **Physical activity** | |  |  |  |  |  |  |  |  |  |
| South | 1624 | 1196 |  | 1181 | 1639 |  | 601 | 1301 | 918 | 2820 |
| West | 901 | 615 |  | 759 | 757 |  | 388 | 712 | 416 | 1516 |
| North | 701 | 651 |  | 588 | 764 |  | 380 | 616 | 356 | 1352 |
| Central | 846 | 1379 |  | 1043 | 1182 |  | 663 | 1029 | 533 | 2225 |
| East | 793 | 1470 |  | 979 | 1284 |  | 632 | 1051 | 580 | 2263 |
| Northeast | 126 | 252 |  | 197 | 181 |  | 82 | 182 | 114 | 378 |
| Total | 4991 | 5563 |  | 4747 | 5807 |  | 2746 | 4891 | 2917 | 10554 |
| **Body Mass Index** | |  |  |  |  |  |  |  |  |  |
| South | 1595 | 1191 |  | 1181 | 1605 |  | 600 | 1280 | 906 | 2786 |
| West | 874 | 613 |  | 763 | 724 |  | 375 | 702 | 410 | 1487 |
| North | 666 | 631 |  | 568 | 729 |  | 364 | 587 | 346 | 1297 |
| Central | 832 | 1352 |  | 1040 | 1144 |  | 636 | 1019 | 529 | 2184 |
| East | 771 | 1448 |  | 982 | 1237 |  | 607 | 1040 | 572 | 2219 |
| Northeast | 127 | 248 |  | 199 | 176 |  | 80 | 181 | 114 | 375 |
| Total | 4865 | 5483 |  | 4733 | 5615 |  | 2662 | 4809 | 2877 | 10348 |
| **Waist circumference** | | |  |  |  |  |  |  |  |  |
| South | 1600 | 1191 |  | 1184 | 1607 |  | 601 | 1282 | 908 | 2791 |
| West | 874 | 614 |  | 764 | 724 |  | 375 | 703 | 410 | 1488 |
| North | 668 | 634 |  | 571 | 731 |  | 365 | 590 | 347 | 1302 |
| Central | 832 | 1357 |  | 1044 | 1145 |  | 636 | 1023 | 530 | 2189 |
| East | 773 | 1453 |  | 985 | 1241 |  | 609 | 1041 | 576 | 2226 |
| Northeast | 127 | 249 |  | 199 | 177 |  | 80 | 182 | 114 | 376 |
| Total | 4874 | 5498 |  | 4747 | 5625 |  | 2666 | 4821 | 2885 | 10372 |
| **Blood pressure** | |  |  |  |  |  |  |  |  |  |
| South | 1638 | 1203 |  | 1200 | 1641 |  | 616 | 1304 | 921 | 2841 |
| West | 908 | 627 |  | 775 | 760 |  | 397 | 716 | 422 | 1535 |
| North | 693 | 645 |  | 580 | 758 |  | 377 | 605 | 356 | 1338 |
| Central | 846 | 1379 |  | 1046 | 1179 |  | 663 | 1031 | 531 | 2225 |
| East | 788 | 1479 |  | 989 | 1278 |  | 636 | 1051 | 580 | 2267 |
| Northeast | 129 | 251 |  | 199 | 181 |  | 83 | 183 | 114 | 380 |
| Total | 5002 | 5584 |  | 4789 | 5797 |  | 2772 | 4890 | 2924 | 10586 |
| **Fasting blood glucose** | | |  |  |  |  |  |  |  |  |
| South | 1451 | 1121 |  | 1087 | 1485 |  | 540 | 1181 | 851 | 2572 |
| West | 792 | 548 |  | 681 | 659 |  | 348 | 621 | 371 | 1340 |
| North | 608 | 590 |  | 530 | 668 |  | 334 | 545 | 319 | 1198 |
| Central | 770 | 1308 |  | 974 | 1104 |  | 609 | 958 | 511 | 2078 |
| East | 693 | 1372 |  | 902 | 1163 |  | 571 | 951 | 543 | 2065 |
| Northeast | 101 | 227 |  | 167 | 161 |  | 66 | 159 | 103 | 328 |
| Total | 4415 | 5166 |  | 4341 | 5240 |  | 2468 | 4415 | 2698 | 9581 |
| **Clustering of risk factors** | | |  |  |  |  |  |  |  |  |
| South | 1428 | 1113 |  | 1077 | 1464 |  | 529 | 1169 | 843 | 2541 |
| West | 774 | 538 |  | 674 | 638 |  | 332 | 610 | 370 | 1312 |
| North | 597 | 576 |  | 522 | 651 |  | 325 | 533 | 315 | 1173 |
| Central | 755 | 1279 |  | 961 | 1073 |  | 581 | 944 | 509 | 2034 |
| East | 684 | 1349 |  | 899 | 1134 |  | 550 | 946 | 537 | 2033 |
| Northeast | 101 | 222 |  | 167 | 156 |  | 64 | 156 | 103 | 323 |
| Total | 4339 | 5077 |  | 4300 | 5116 |  | 2381 | 4358 | 2677 | 9416 |
| **Ten-Year CVD risk (40-69 Years)** | | | |  |  |  |  |  |  |  |
| South | 598 | 425 |  | 433 | 590 |  | NA | 364 | 659 | 1023 |
| West | 255 | 175 |  | 232 | 198 |  |  | 162 | 268 | 430 |
| North | 221 | 165 |  | 182 | 204 |  |  | 145 | 241 | 386 |
| Central | 267 | 328 |  | 299 | 296 |  |  | 238 | 357 | 595 |
| East | 255 | 456 |  | 337 | 374 |  |  | 300 | 411 | 711 |
| Northeast | 35 | 73 |  | 62 | 46 |  |  | 36 | 72 | 108 |
| Total | 1631 | 1622 |  | 1545 | 1708 |  |  | 1245 | 2008 | 3253 |
|  |  |  |  |  |  |  |  |  |  |  |

Abbreviations: CVD: Cardiovascular diseases; NA- Not Applicable; CI: Confidence Interval
